# Population admixture and high larval viability among urban toads

**DOI:** 10.1002/ece3.578

**Published:** 2013-05-02

**Authors:** Kazuko Hase, Naruo Nikoh, Masakazu Shimada

**Affiliations:** 1 Department of General Systems Studies, The University of TokyoMeguro, Tokyo, 153-8902, Japan; 2 Department of Liberal Arts, The Open University of JapanChiba, 261-8586, Japan

**Keywords:** Genetic disturbance, introgressive hybridization, population admixture, urban

## Abstract

In terms of evolutionary biology, a population admixture of more than two distinct lineages may lead to strengthened genetic variation through hybridization. However, a population admixture arising from artificial secondary contact poses significant problems in conservation biology. In urban Tokyo, a population admixture has emerged from two lineages of Japanese common toad: native *Bufo japonicus formosus* and nonnative *B. japonicus japonicus*, of which the latter was introduced in the early 20th century. To evaluate the degree of genetic disturbance in the admixed population of these two subspecies, we analyzed genotypes of toads distributed within and outside Tokyo by assessing mtDNA and seven microsatellite loci. We found that the introduced *B. japonicus japonicus* genotype dominates six local populations in the Tokyo admixture zone and was clearly derived from past introgressive hybridization between the two subspecies. These observations were supported by morphological assessments. Furthermore, the average larval survival rate in Tokyo was significantly higher than that outside Tokyo, suggesting that the temporary contribution of introduced toads occurred through introgression. The fitness of toads in urban Tokyo may thus be increasing with the assistance of nonnative individuals.

## Introduction

Population admixture is an important topic in evolutionary biology because it offers the advantage of local adaptation by increasing genetic variation through hybridization within and between species (Song et al. 2011). Recently, hybridization between geographically isolated populations has attracted attention as an important speciation process in plant and animal taxa (Bullini and Nascetti 1990; Barton 2001; Mallet 2007). Introgressive hybridization that occurs in combination with population admixture sometimes produces new recombinant genotypes with enhanced genetic diversity; it also sometimes produces partial genetic incompatibilities that create a problem for the long-term persistence of the population. If the survival rate of the admixed population can be sustained, the divergence of hybrid offspring from the parental lineages could be increased (Templeton 1981; Dowling and Secor 1997).

Population admixture can be classified into two types: admixture of two or more distinct lineages in a natural contact zone and admixture of native and introduced lineages because of human-mediated secondary contact.

Genetic admixture of two or more divergent populations is more common in the wild than was previously comprehended by biologists (Mallet 2007). Several hybridization events between divergent species in secondary contact zones have been documented under natural conditions in, for example, anurans (McDonnell et al. 1978; Gartside et al. 1979; Lamb and Avise 1986; Parris 2001; Pfennig 2007). In contrast, anthropogenic conditions cause several serious problems. Some introduced species successfully adapt to an artificial environment and spread throughout the habitat overcoming the native species (Tait et al. 2005; Wania et al. 2006; Niinemets and Penuelas 2008). This often involves hybridization with natives and poses an unpredictable threat to local ecosystems (Trusty et al. 2007; Ryan et al. 2009).

Nowadays more than 50% of the human population lives in urban areas (UNDP 2010). Urbanization has a dramatic influence on ecosystems and is one of the most serious concerns in conservation biology (McKinney 2002; Hamer and McDonnell 2008). Habitat loss and fragmentation due to increased urbanization have negative impacts such as reductions in population size, loss of connectivity between subpopulations, reduced genetic diversity, bottlenecks, and inbreeding suggesting a heightened risk of local extinction (Hitchings and Beebee 1997, 1998; Andersen et al. 2004; Cushman 2006; Noel et al. 2007; Dixo et al. 2009). In this context, the introduction of individuals from another lineage and the establishment of population admixture could help in maintaining genetic diversity. In particular, when conservation objectives are very context dependent, admixture could be beneficial for conservation. However, this approach is complicated because there is a risk of losing distinct genetic components of the original population that are well adapted to the local environment. Thus, population admixture due to artificial introduction can cause genetic disturbance through hybridization between native and introduced species.

Despite the presence of numerous introduced species in urban areas, few empirical studies of genetic disturbance, that is, of the introduction of species that cause native species to lose their original genetic components through introgression have been performed aside from studies of some plants (Ellstrand and Schierenbeck 2000). It is difficult to assess the degree of invasiveness of hybrids, the potential to establish an ecological niche, because even if F1 hybrids have no heterosis, the hybrid genotype can confer higher fitness in later generations. First or early-generation hybrids sometimes show outbreeding depression due to a disadvantage in heterozygosity or genetic incompatibility, which leads to lower fitness. However, recent long-term studies on the effect of hybridizations revealed that few strains of hybrid in a population can recover outbreeding depression and produce higher fitness in F2 and later generations than parental (Erickson and Fenster 2006; Hwang et al. 2011; Szűcs et al. 2012). These are really complicated problems in urban ecology. Nevertheless, the investigation of biological phenomena under artificial conditions can provide biologists with as much insight as research conducted under natural conditions. Various biological (microevolution) processes can be observed in real-time as populations pass through each ecological phase, which can also provide us with valuable knowledge that can be applied for conservation purposes. Ecologists should fairly evaluate the roles of species in their environment regardless of their origins (Davis et al. 2011), even in artificial urban environments.

**Figure 1:**
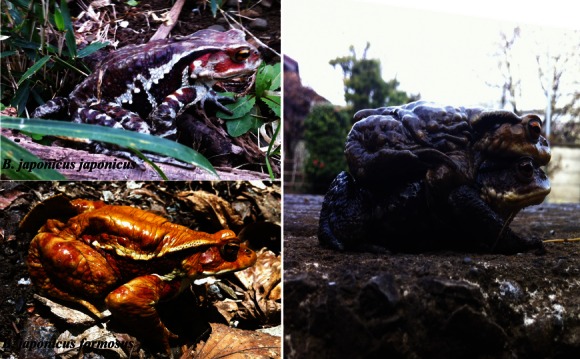
Feature of the two subspecies of the Japanese common toad: *Bufo japonicus japonicus* (small tympanum) and *B. japonicus formosus* (large tympanum), and a breeding pair of Tokyo area that has almost similar *B. japonicus japonicus* features.

In this article, we evaluated genetic disturbance (the degree of introgression from introduced nonnative *Bufo japonicus japonicus* to native *B. japonicus formosus*) and its effect on the fitness of an admixed population of *B. japonicus* in urban Tokyo. Two divergent lineages of the Japanese common toad are found in urban Tokyo: the native *B. japonicus formosus* and the nonnative *B. japonicus japonicus*, which was artificially introduced in the early 20th century. These two subspecies of toads have established admixed local populations (Hase et al. 2012). *Bufo japonicus japonicus* and *B. japonicus formosus* are naturally distributed in the western and eastern regions of mainland Japan, respectively (Matsui 1984). Both subspecies possess 22 diploid chromosomes (presumably with the ZZ/ZW system of sex determination; inferred from related species) (Ponse 1942; Kawamura et al. 1980; Matsui et al. 1985; Miura 1995), and they have the same ecological and morphological features except that the tympanum of *B. japonicus formosus* is approximately twice as large as that of *B. japonicus japonicus* (Matsui 1984; Fig. 1). Phylogenetic analysis based on mitochondrial DNA (mtDNA) Cyt*b* indicated that the species *B. japonicus* was clearly divided into two lineage clades: Western *B. japonicus japonicus* and Eastern *B. japonicus formosus*, which diverged approximately 5.7 million years ago (Igawa et al. 2006). To assess genetic disturbance, we analyzed the genetic structure of toads in three distinct groups based on mtDNA lineages: native *B. japonicus japonicus* from western Japan (Western), native *B. japonicus formosus* from eastern Japan (Eastern), and an admixture zone containing both lineages in urban Tokyo (Tokyo). The genetic structure was investigated by multilocus genotype based on mtDNA and seven microsatellite loci. Simultaneously, to support the genetic data, we compared adult morphologies among the three distinct groups using a morphometric index standardized by Matsui (1984). Furthermore, to study the effect of genetic disturbance on fitness, we evaluated and compared the viability of larvae by monitoring their survival and rate of development in each of the three groups.

## Materials and Methods

### Study site and DNA sampling

**Figure 2:**
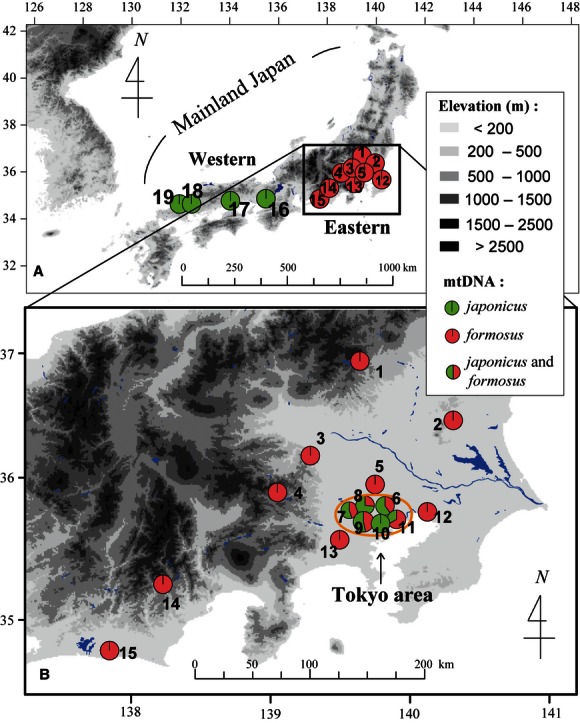
Map of sampling sites with local population mtDNA lineages. (A) Geographical overview of mainland Japan. (B) Enlarged details for eastern Japan and the Tokyo area. Local populations were divided into three groups in this study: Eastern group, Pops. 1–5 and 12–15 with the *formosus* mtDNA lineage; Western group, Pops. 16–19 with the *japonicus* mtDNA lineage; Tokyo group, Pops. 6–11 with both *japonicus* and *formosus* mtDNA lineages.

Sampling was conducted in and around breeding ponds between 2008 and 2011 during the breeding season at 19 sites across the eastern and western regions of mainland Japan. The collection locality (population sites) and the sample attributes are shown in Figure 2 and Table 1. We collected 293 specimens of *B. japonicus* (including 75 specimens that were used in Hase et al. 2012) from three districts: the eastern region of Japan – native *B. japonicus formosus* (Eastern group: Pops. [local populations] 1–5 and 12–15); the western region of Japan – native *B. japonicus japonicus* (Western group: Pops. 16–19); and the urban Tokyo area – an admixture of *B. japonicus formosus* and *B. japonicus japonicus* (Tokyo: Pops. 6–11). Populations 1, 6–11, 14, and 17 were from single ponds and Populations 3, 4, and 18 were from three, two, and four small pools, respectively. To avoid gathering only kin, larvae were randomly selected from each pond and all individuals were released after the larval tails or toe tips of the adults and juveniles were collected.

**Table 1:** Taxonomy, group, locality of population sites, and number of Japanese common toad samples from embryos, larvae, juveniles, and adults

Pop. ID	Taxon	Group	Locality	Coordinates	Individuals	Embryos	Larvae	Juveniles	Adults	Male	Female
1	*Bufo japonicus formosus*	Eastern	Nikko, Tochigi prefecture	N36.7513°E139.5855°	31		29^1^		2	1	1
2			Ushiku, Ibaraki prefecture	N36.3184°E140.3015°	2				2	1	1
3			Yorii, Saitama prefecture	N36.1155°E139.2201°	15		11		4	3	1
4			Chichibu, Saitama prefecture	N35.8877°E138.8114°	8		8				
5			Niiza, Saitama	N35.7897°E139.5609°	14		14^1^				
12			Makuhari, Chiba	N35.6592°E140.0588°	1				1^2^	–	–
13^3^			Zama, Kanagawa	Unknown	12				12	12	–
14			Abeoku, Shizuoka	N35.3024°E138.3410°	10		10				
15^3^			Hamamatu, Shizuoka	Unknown	2				2	–	2
6	*B. japonicus*	Tokyo	Bunkyo, Tokyo	N35.7125°E139.7228°	25		25^1^				
7			Fuchu, Tokyo	N35.6911°E139.4669°	10		10^1^				
8			Mitaka, Tokyo	N35.6800°E139.5298°	15	1^2^	14^1^				
9			Chofu, Tokyo	N35.6582°E139.5302°	32	3^2^	11^1^	6^1^	12^1^	11^1^	1
10			Komaba, Tokyo	N35.6592°E139.6879°	27		22^1^		5^1^	–	–
11			Shibuya, Tokyo	N35.6574°E139.7068°	24	2^2^	22^1^				
16^3^	*B. japonicus japonicus*	Western	Arashiyama, Kyoto	Unknown	5				5	3	2
17			Mt. Azuma, Hiroshima	N35.0646°E133.0272°	20		16		4	4	
18			Yoshiwa, Hiroshima	N34.4570°E132.1318°	32		25		7	2	5
19^3^			Yoshiwa, Hiroshima	Unknown	8				8	7	1
	Total				293		223	6	64	44	14

Sexes were only determined in a subset of adults.

1 Individuals partly including the same samples as Hase et al.(2012).

2 The same samples as Hase et al. (2012).

3 The collections of Institute for Amphibian Biology, Hiroshima University, which were consisted of adult individuals captured in 1977 to 1983 from native habitat defined as each local population site.

### DNA extraction and microsatellite amplification

For the samples collected in 2008–2009, total DNA was extracted from tissues using a Qiagen DNeasy Blood and Tissue Kit (Qiagen, Inc., Valencia, CA). For the 2010–2011 samples, the tail tips of the larvae and the toe tips of the adults were digested overnight in a solution (0.3% sodium dodecyl sulfate [SDS], 400 mmol/L NaCl, 5 mmol/L ethylenediaminetetraacetic acid [EDTA], 20 mmol/L Tris–HCl, pH 8.0) containing 200 μg/mL proteinase K at 55°C.

We selected seven microsatellite primer pairs developed for *Bufo bufo*: *Bbufu11*, *Bbufu13*, *Bbufu23*, *Bbufu39*, *Bbufu49*, and *Bbufu62* (Brede et al. 2001). In addition, due to the existence of a few mutations, we created locus *Bjap14* by designing *Bbufu14* (Brede et al. 2001). The primer pair for *Bjap14*, Bjap14F (5′-GCGTGTGTACATGAGGAATAACA-3′), and Bjap14R (5′-AGTGAGCAGGAGCTGAGGAG-3′) were designed inside the *Bbufu14* fragment to include the microsatellite with confirmational sequencing. *Bbufu14* fragments were amplified by 30 cycles of polymerase chain reaction (PCR) under the following conditions: denaturation at 94°C for 30 sec, annealing at 55°C for 60 sec, and extension at 72°C for 20 sec. PCR was performed using Ampdirect plus PCR buffer (Shimadzu, Inc., Kyoto, Japan) and ExTaq (TaKaRa Bio, Inc., Shiga, Japan) in a final volume of 10 μL. TA cloning was performed using the pGEM-T vector system (Promega, Inc., Madison, WI) for ligation and XL10-Gold Ultracompetent Cell (Stratagene, Inc., La Jolla, CA) for transformation. The inserted fragments were sequenced using nested vector primers T7 and Sp6 (Promega). The *Bjap14* sequence was deposited in the GenBank database (Acc. no. AB713497). PCR conditions for these seven microsatellite loci are detailed in Table A1.

The fragment size of the PCR products from each microsatellite locus was analyzed using a CEQ8000 Genetic Analysis System (Beckman Coulter, Inc., Brea, CA) with a Genomelab Size Standard Kit 400 (60–420 bp, Beckman Coulter).

### Analyses of mitochondrial lineages

We investigated mtDNA from all DNA samples. A 0.8-kb segment of the mtDNA Cyt*b* gene was amplified by PCR from the samples and subjected to restriction fragment length polymorphism (RFLP) genotyping using restriction endonuclease SalI, which cuts the fragment of the *B. japonicus formosus* Cyt*b* gene into 0.2 kb and 0.6 kb lengths by digestion; DNA sequencing was conducted as described previously (Hase et al. 2012). All mitochondrial sequences reported were deposited in the GenBank databases (Acc. nos. AB597912–AB597929 from Hase et al. 2012; AB713498–AB713517). The phylogenetic relationships were inferred by the maximum likelihood (ML) and Bayesian inference (BA) methods. Sequence alignments and selection of the best model of nucleotide substitution for the phylogenetic analysis were performed using MEGA 5.0 (Tamura et al. 2011). The ML tree was estimated using the GTR + Γ model method in MEGA 5.0 (Tamura et al. 2011) and support values for the internal nodes were inferred from 1000 bootstrap replicates. For the BA analysis, we used the program MrBayes 3.2.0 (Ronquist and Huelsenbeck 2003). A total of 50,000 trees were obtained and the first 25,000 trees were considered “burn-in” and discarded (model: GTR + Γ; 2.5 M generations; sample freq = 100). The posterior probability of each node was used as the support value of the node.

### Genetic variations and structure

We used MICROCHECKER v. 2.2.3 to identify genotyping errors and null alleles (Van Oosterhout et al. 2004). We tested the genotypic linkage disequilibrium between each pair of loci in each local population using ARLEQUIN v. 3.5 (Excoffier and Lischer 2010). Significance was adjusted for multiple tests using Bonferroni corrections. The number of alleles and observed (H_O_) and expected (H_E_) heterozygosities for each microsatellite marker within the samples were calculated using Genepop v. 4.0 (Raymond and Rousset 1995). To calculate the mean number of alleles, allelic richness, H_E_, and *F*_IS_ across loci for each local population, we used FSTAT v.2.9.3 (Goudet 1995). We calculated the pair-wise *F*_ST_ among all local populations (for more than eight samples) and its significance value was obtained by a permutation test (17,100 permutations) using FSTAT v.2.9.3 (Goudet 1995). Isolation by distance was tested with a Mantel test (10,000 permutations) by regressing pair-wise *F*_ST_/(1 − *F*_ST_) against the shortest geographic distance (km) (Rousset 1997) using IBD software (Bohonak 2002). To detect recent population bottlenecks, we ran the program BOTTLENECK (Cornuet and Luikart 1996) using a two-phase model with 90% stepwise mutation. Significance was assessed using Wilcoxon's Signed Rank test.

In order to assess genetic differentiation among the three groups, namely the Western (*B. japonicus japonicus*), Eastern (*B. japonicus formosus*), and Tokyo (admixture) groups, we implemented an analysis of molecular variance (AMOVA) of seven microsatellite loci based on the *F*_ST_ value in ARLEQUIN v.3.5 (Excoffier and Lischer 2010). The statistical significance of the variance components of the microsatellite loci was evaluated using 10,000 random permutations. To investigate past introgressive hybridization between *B. japonicus japonicus* and *B. japonicus formosus* in the Tokyo area, we assessed the genetic structure of the microsatellite data of our sample set across the three groups using the Bayesian clustering method implemented in STRUCTURE v. 2.2 (Pritchard et al. 2000). We used an admixture model with correlated allele frequencies between populations (genetic clusters). Simulations were performed with 10 runs for each proposed *K*-value of 1–20 (the number of sampling sites). The length of the burn-in period was set to 50,000 iterations followed by 100,000 Markov chain Monte Carlo repetitions. To identify the best genetic cluster value (*K*) for our data set, we applied the method for the Δ*K* statistic described by Evanno et al. (2005).

### Morphometric assessment

In a previous observational study (Matsui 1984), a total of 2525 *B*. *japonicus* individuals from 96 populations were analyzed to determine morphometric clines in relation to geographical and/or climatological parameters. The findings from this study indicated that there is a clear difference in the morphology among the populations because *B*. *japonicus formosus* has tympanums that are approximately twice as large as those of *B*. *japonicus japonicus*.

To assess hybridization of the toads in the Tokyo admixed population through morphometrics, we compared a morphological index standardized in the previous study (Matsui 1984): the diameter of the tympanum per tympanum–eye distance. In spring 2011, we measured the morphological parameters of 30 individual adult toads from the three groups: Eastern (*B. japonicus formosus*), 12 adults from Pop. 1 (five males and one female), Pop. 2 (one male and one female), and Pop. 3 (three males and one female); Western (*B. japonicus japonicus*), eight adults from Pop. 17 (two males) and Pop. 18 (three males and three females); and Tokyo (admixture), 10 males from Pop. 9. All individuals were determined to be *B. formosus* or *B. japonicus* based on their mtDNA assessed through the above-mentioned RFLP method.

### Monitoring of survival and development

To evaluate the influence of genetic characteristics on biological fitness among the three groups, we monitored the survival and development of the larvae from March to May in 2010 and 2011 in our laboratory. We monitored 25 cohorts in total: Eastern, seven cohorts from Pop. 1 (*N* = 3), Pop. 3 (1), Pop. 5 (2), and Pop. 14 (1); Western, six cohorts from Pop. 17 (3) and Pop. 18 (3); and Tokyo, 13 cohorts from Pop. 6 (1), Pop. 8 (1), Pop. 9 (4), Pop. 10 (4), and Pop. 11 (2). We sampled 0.3–0.4 m spawn strings laid by one female and treated larvae hatched from one string as a unit of cohort. Each spawn string from each population locality was transferred to a separate plastic tank in the laboratory and left until hatching was confirmed by the observation of spawn protruding from the string. To maintain equality of larval density, 50 embryos per cohort were randomly chosen and transferred to a container (W220 × D310 × H40 mm) with 1.2 L dechlorinated tap water. The larvae were raised with a sufficient supply of fish pellets in a large incubator at 18°C with a 12:12-h dark:light cycle. Survivors were counted every day for 40 days. The survival rate was defined as the number of hatched survivors of each cohort that developed forelimbs (Gosner Stage ≥42; Gosner 1960) by day 40, which allows enough time for the development of forelimbs and for changing to pulmonary respiration from gill-based respiration. The larvae of toads (Bufonidae) usually develop in phase in schools, and freshly metamorphosed juveniles move onto land together at as high a density as possible. Because the life history stage of toads that has the highest mortality is that after landing, a high larval survival rate is of great importance when assessing fitness (cf., Miyamae and Matsui 1979; Goater 1994). Finally, 1–4 of the surviving larvae from some randomly chosen cohorts were genotyped and added to the samples for each local population.

We compared Kaplan–Meier survival curves for the populations with log-rank tests using R 2.15.1 (R Development Core Team 2009). To analyze the influence of group differentiation on larval viability (survival rate at day 40), we used a generalized linear mixed model (GLMM) with binomial error distribution and a log-link function using the glmmML package in R 2.15.1 (R Development Core Team 2009). The best model is selected by Akaike's information criterion (AIC) score. We treated the groups (Western, Eastern, and Tokyo) as fixed variables and individuals of each cohort as a random variable. Furthermore, to clarify the effects of different mtDNA lineages (*japonicus* and *formosus*) on larval viability within the Tokyo group, we compared Kaplan–Meier survival curves between the two lineages and performed a second analysis with a GLMM treating mtDNA lineage as a random variable.

## Results

### Mitochondrial lineages of the population groups

The population locality and mtDNA lineage of individuals from each local population are shown in Figure 2.

**Figure A1:**
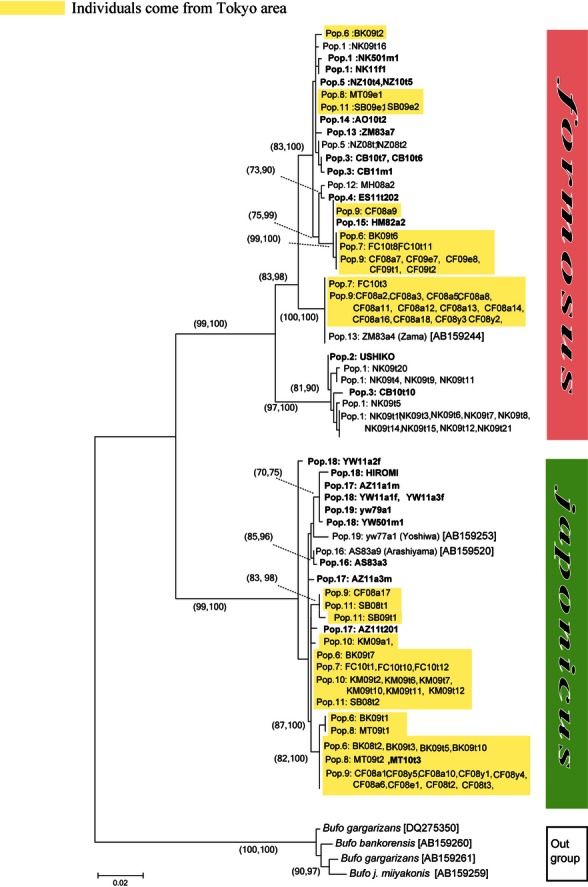
Maximum likelihood (ML) tree based on 831 bp of Cytb sequences. The Bayesian inference (BA) tree exhibited similar topologies. The values (over 70%) of the bootstrap support for the ML method and the posterior probability for the BA method are shown in parenthesis. The population numbers (Pops. 1_19) of the specimens correspond to the sampling sites (as in Fig. 1 and Table 1). Specimens with newly determined sequences are shown in bold. All individuals belonging to the Western group (Pops. 16_19) were included in the japonicus clade and all individuals belonging to the Eastern group (Pops. 1_5, 13_ 15) were included in the formosus clade. However, as reported in Hase et al. (2012), the Tokyo area (Pops. 6_11) consisted of both japonicus and formosus clades. While only Pop. 10 consisted of the japonicus clade, other populations in Tokyo (Pop. 6_9 and 11) possessed both japonicus and formosus clades.

Our previous phylogenetic analysis revealed that Japanese common toads in the Tokyo area have two major mitochondrial lineage types: Western and Eastern, which correspond to *B. japonicus japonicus* (hereafter, *japonicus*) and *B. japonicus formosus* (ditto, *formosus*), respectively (Hase et al. 2012). These findings are consistent with the two lineage patterns identified by RFLP genotyping. All 65 individuals from Pops. 16–19 (Western group) had the *japonicus* lineage pattern and all 95 individuals from Pops. 1–5 and Pops. 12–15 (Eastern group) had the *formosus* lineage pattern. In contrast, individuals sampled in the Tokyo area had a mixture of *formosus* and *japonicus* lineage patterns: 10 *formosus* and 15 *japonicus* in Pop. 6; five *formosus* and five *japonicus* in Pop. 7; four *formosus* and 11 *japonicus* in Pop. 8; 15 *formosus* and 17 *japonicus* in Pop. 9; 27 *japonicus* in Pop. 10 (located at our university campus and considered a source of the alien introduction); 16 *formosus* and eight *japonicus* in Pop. 11. Molecular phylogenetic analysis of Cyt*b* sequences was performed together with previously reported analyses (Igawa et al. 2006; Hase et al. 2012). The phylogenetic analysis strongly supported the above-mentioned results and the findings are detailed in Figure A1.

### Genetic variation based on microsatellite analyses

We successfully amplified all seven microsatellite loci in our sample. According to MicroChecker, several null alleles were detected at low frequencies in four local populations for locus *Bbufu13* (Pops. 1, 13, 17, and 18), in three local populations each for loci *Bbufu23* (Pops. 1, 6, and 13) and *Bbufu39* (Pops. 1, 6, and 10), in two local populations for locus *Bbufu11* (Pops. 10 and 11), and in one local population for locus *Bbufu62* (Pop. 11). Significant deviations from genotypic linkage equilibrium were detected in 55 of 378 tests (after sequential Bonferroni correction, data not shown). However, no overall deviation was detected across all local populations. The mean number of alleles, allelic richness, and H_E_ across loci for each local population ranged from 2.0 to 5.0, 1.50 to 3.74, and 0.40 to 0.57, respectively (detailed in Table A2). Ten populations showed significantly positive F_IS_ values (range: 0.145–0.402; Table A2).

Our sampling design (pooling kin larvae) must have resulted in some heterozygote deficits, but no consistent locus was detected in the null allele frequencies and there was no deviation from Hardy–Weinberg equilibrium across the local populations. Therefore, we retained all seven loci for the subsequent analyses. In addition, despite the low level of genetic variation in many of the local populations studied, no significant recent bottleneck was detected by the program BOTTLENECK (Cornuet and Luikart 1996).

### Population structure and differentiation

**Figure A2:**
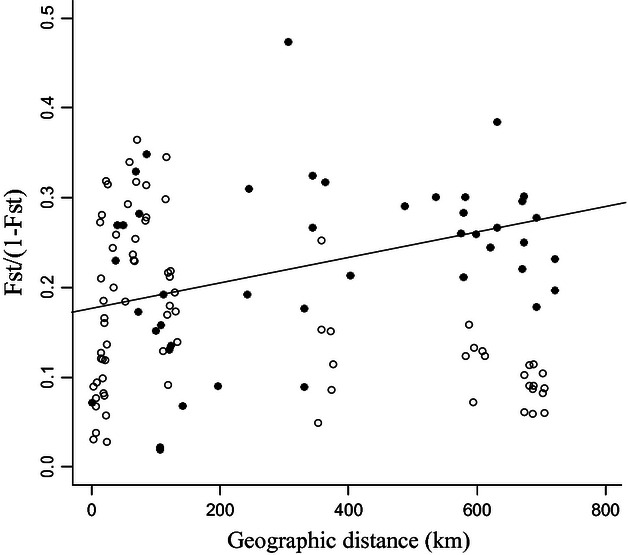
Relationship between pairwise genetic distance and pairwise geographic distance in local populations of Bufo japonicus japonicus and B. japonicus formosus. Open circles indicate the relationship between all populations and closed circles (solid regression line) indicate the relationship between local populations except for in the Tokyo area. The slope of the regression line was significantly greater than zero (10,000 bootstrap randomizations): FST/(1 – FST) = 0.1770024 + 0.001412 [geographic distance]; r = 0.37, P = 0.01 among all populations except in theTokyo area.

According to the AMOVA analysis, the highest level of differentiation was within local populations (17.05% of variation), the next highest was among local populations within groups (9.18%), and the lowest was among groups (7.01%) (Table A3). The highest significant pair-wise *F*_ST_ value was 0.28 (Pops. 4 and 19) and the lowest was 0.04 (Pops. 6 and 11) (Table A4). Except for in the Tokyo area, genetic differentiation among local populations (pair-wise *F*_ST_/[1 − *F*_ST_]) significantly reflected geographic distance (*r* = 0.37, *P* = 0.01) (Fig. A2).

**Figure 3:**
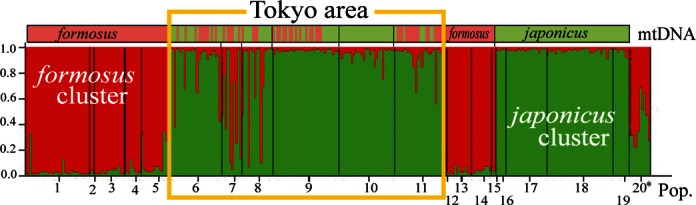
Probabilistic assignment to genetic clusters based on Bayesian analyses. Graphical output from STRUCTURE for *K* = 2 based on the seven microsatellite markers for the Japanese common toads belonging to Pops. 1–19 and Pop. 20* (represents the hybrids between one female *B. japonicus japonicus* from Pop. 18 and one male *B. japonicus formosus* from Pop. 1 [K. Hase, unpubl. data]).

The Bayesian clustering method implemented in STRUCTURE (Pritchard et al. 2000) showed the highest likelihoods (Δ*K*_max_ = 163.6) for the model with *K* = 2 as the optimum number of genetic clusters for our dataset (Fig. S1). All local populations were divided into two genetic clusters, a *japonicus* cluster and a *formosus* cluster, which approximate the two mtDNA lineages (Fig. 3). At the group level, all individuals from the Western group (Pops. 16–19) were assigned to the *japonicus* cluster with probabilities higher than 90%. In contrast, almost all the individuals from the Eastern group were assigned to the *formosus* cluster with probabilities higher than 90%; whereas, some of these had low (<90%) probabilities, including two individuals from Pop. 1, one individual from Pop. 4, and four individuals from Pop. 5. One individual from Pop. 12 that possessed the *formosus* mtDNA lineage was assigned with 95% probability to the *japonicus* cluster (Fig. 3).

Local populations in Tokyo (Pops. 6–11 in Table 1 and Fig. 3), an admixture zone between the two mtDNA lineages, showed a more complicated pattern. Although 107 of the 133 individuals were assigned to the *japonicus* cluster with probabilities higher than 90%, only three of the 133 individuals were assigned to the *formosus* cluster with probabilities higher than 90%. Several individuals from Tokyo were assigned to one cluster (40 to *japonicus* and one to *formosus*) with probabilities higher than 90%, but possessed the opposite mtDNA lineage. Twenty-two individuals from Tokyo showed low (<90%) assignment probabilities for both the *japonicus* or *formosus* clusters.

**Figure 4:**
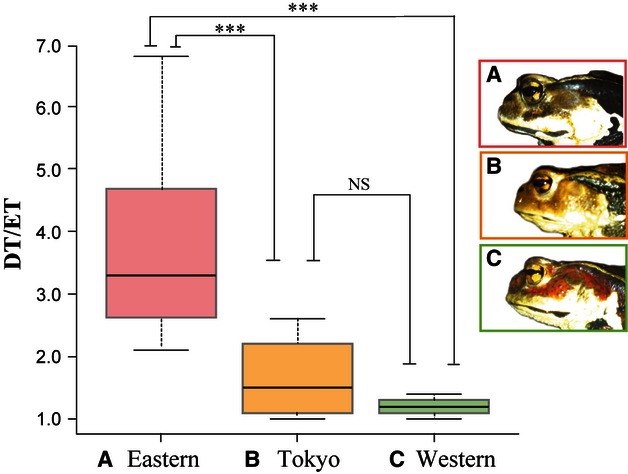
Comparison of morphological values based on Matsui (1984): DT/ET = the diameter of the tympanum relative to the tympanum–eye distance. Each group consisted of native adult toads: (A) Eastern (*Bufo japonicus formosus*) from Pops. 1, 2, and 3 (*N* = 12, median = 3.30, mean = 3.81 ± SE 0.13); (B) Tokyo from Pop. 9 (*N* = 10 [mtDNA type *japonicus*: *n* = 3; and *formosus*: *n* = 7], median = 1.50, mean = 1.60 ± SE 0.06); and (C) Western (*B. japonicus japonicus*) from Pops. 17 and 18 (*N* = 8, median = 1.20, mean = 1.20 ± SE 0.02). The photograph of three adult toads shows (A) Eastern, (B) Tokyo, and (C) Western toads, respectively. The body color shows natural variation.

Figure 4 shows a comparison of morphology among the Western (mtDNA = *japonicus*), Eastern (*formosus*), and Tokyo (both) (Pop. 9) groups based on a previous study (Hase et al. 2012). The morphological index was significantly different among the three groups (Kruskal–Wallis one-way analysis of variance by rank, *K* = 21.92, *P* = 0.00002). The Eastern and Western groups had significantly different values (*P* = 0.0005; Steel–Dwass test). The Tokyo group was also significantly different from the Eastern group (*P* = 0.00004; Steel–Dwass test). There were no significant differences between the Tokyo and Western groups.

### Comparison of larval survival

**Figure 5:**
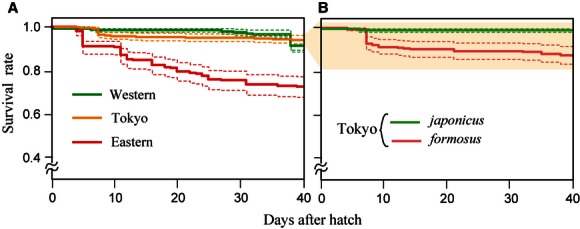
Survival curves for Japanese common toad larvae over 40 days after hatching. The values of Kaplan–Meier plots are shown with respect to (A) each group and (B) two mtDNA lineages within Tokyo group. Dashed lines represent 95% confidence intervals.

The Kaplan–Meier survival curves for the larvae from the three groups during the 40 days after hatching are shown in Figure 5. Although there were significant differences between the survival curves of the Eastern and Tokyo (χ^2^ = 76.7, df = 1, *P* < 0.0001 [Bonferroni adjustment]) and Eastern and Western (χ^2^ = 42.8, df = 1, *P* = 0.0003 [Bonferroni adjustment]) groups, there was no significant difference between the Western and Tokyo groups (Fig. 5A). The average survival rate at day 40 in the Western, Eastern, and Tokyo groups was 92.7% (±SE 1.55), 73.4% (±SE 3.90), and 93.2% (±SE 0.66), respectively (more details on the monitoring experiments can be found in Table S1). GLMM supported a significant relationship between the survival rates of the Eastern and Tokyo groups (*z* = 2.692, *P* = 0.0071), whereas there were no significant relationship between the survival rates of the Western and Eastern and Western and Tokyo groups. The effects of mtDNA lineages on survival rate within Tokyo group (average of *japonicus* and *formosus* were 99.4% [*N* = 7, ±SE 0.22] and 83.2% [*N* = 5, ±SE 5.48], respectively) were demonstrated both by the Kaplan–Meier survival curves (Fig. 5B; χ^2^ = 42.3, df = 1, *P* = 0.0003) and analysis of GLMM, which indicated that the *japonicus* lineage had a higher survival rate than the *formosus* lineage (*z* = 2.105, *P* = 0.035).

## Discussion

Unlike previous reports of amphibians in urban areas (Hitchings and Beebee 1997, 1998), we found no decrease in genetic diversity or larval viability. However, the population admixture was obtained by an artificial process with serious problems: the introduction of numerous nonnative toads must have resulted in higher genetic diversity and increased larval viability, whereas the situation has deprived native toads of any opportunity to increase genetic or phenotypic diversity of pure lineages.

### Introgression as a genetic disturbance

The results of the Bayesian clustering support our working hypothesis of a past introgressive hybridization between the invasive *B. japonicus japonicus* and the native *B. japonicus formosus* in the Tokyo area (Fig. 3).

Genetic compatibility between two divergent lineages facilitates natural hybridization. A long time is usually required for amphibians to produce mechanisms of reproductive isolation; the divergence time based on the average genetic distance with mtDNA Cyt*b* of congeneric species is estimated to be 7.0 Mya (Johns and Avise 1998), which suggests the possibility of natural hybridization between two species with a genetic distance less than that. Postzygotic reproductive isolation of the genus *Bufo* (92 species not including *B. japonicus*) requires relatively high levels of divergence because the genetic distance (mtDNA fragment 12S–16S) is more than 0.08 (range: 0.002–0.152, mean ± SE = 0.083 ± 0.001) (Malone and Fontenot 2008). However, under natural conditions, the amount of time needed to produce partial genetic incompatibilities that can generate reproductive barriers between relative toads is not as long as suggested by predictions from artificial (laboratory) cross experiments (Colliard et al. 2010). Therefore, we cannot conclude that there was no genetic incompatibility and no negative effect on an evolutionary time scale in the admixed population of *B*. *japonicus japonicus* and *B*. *japonicus formosus* in Tokyo. In fact, we observed lower hatching rate and larval viability in F1 hybrids in a pair crossing experiment between these two subspecies (K. Hase, unpubl. data), which suggests possibility of existing outbreeding depression in early generations of hybrids. Because the situation is particular to Tokyo, it is too early to come to any conclusion that estimated divergence time of 5.7 Mya for *B*. *japonicus japonicus* and *B*. *japonicus formosus* (Igawa et al. 2006) appears too short to produce genetic incompatibilities. Anthropogenic landscapes can lead to hybridization between native and introduced species (Hoban et al. 2012), and the unusual conditions of the Tokyo area may have easily led to the production of hybrids.

### What has driven genotype displacement from native to introduced toads?

In many cases, our clustering analyses of the admixed populations in urban Tokyo indicated that genotype displacement from native *B. japonicus formosus* to introduced *B. japonicus japonicus* had occurred (Fig. 3). But why had introgression not occurred in the opposite direction?

Fauvergue et al. (2012) noted that the establishment success of an introduced small population depends on demographic and environmental stochasticities, Allee effects, genetic drift, inbreeding, selection, adaptation, and relationships among these factors. We considered two possible causes. First, demographic factors: high mtDNA haplotype diversity and the positive Tajima's *D* value reported by Hase et al. (2012) suggest that on one occasion, the native *B. japonicus formosus* population had dramatically decreased in size in the Tokyo area. Although the factor with the greatest influence on our results has not been elucidated, some relationship between demographic and selection factors may have driven the genotype displacement. Approximately 100 years ago, an unknown number of western toads (*B. japonicus japonicus*) were artificially introduced into Tokyo for commercial and experimental (laboratory animal in physiology at university) uses (Hase et al. 2012). Considering that male and female *B. japonicus formosus* attain sexual maturity at the ages of 2–3 and 3–4 years, respectively (Hisai 1981), approximately 30 generations have passed since introgressive hybridization began. Several studies have indicated that rapid displacement of native genotypes by nonnative genotypes can occur in less than 10 generations (Huxel 1999; Epifanio and Philipp 2000; Wolf et al. 2001), which suggests that the introduced *B. japonicus japonicus* could have already spread their genotype throughout the local populations.

Second, genetic factors and their selective forces: genotypic admixture between the two subspecies has contributed to the avoidance of inbreeding depression and has instead increased genetic variation through introgressive hybridization. In plant species, several reports have propounded similar arguments and have shown that multiple introductions from different source populations can provide fitness benefits to an introduced population through the resulting genetic diversity (Lee 2002; Prentis et al. 2008; Verhoeven et al. 2011). Habitat loss and fragmentation caused by urbanization lead to decreased population sizes and lower genetic diversity, and consequently, to genetic drift and inbreeding depression, which can result in local extinction (Gilpin and Soulé 1986; Young et al. 1996; Saccheri et al. 1998; Reed and Frankham 2003). However, with respect to the larval viability (average survival rates), *B. japonicus* had significantly higher values in urban Tokyo than an area outside Tokyo (Eastern group) (GLMM: *z* = 2.692, *P* = 0.0071; Fig. 5A). According to the analysis of the larval survival curves and rates, the difference in mtDNA lineages had the most critical effect on larval viability, that is, the survival curves showed difference between Western (*japonicu*s lineage) and Eastern (*formosus* lineage) groups (log-rank test: *P* = 0.0003; Fig. 5A) and the *japonicus* lineage had higher viability than the *formosus* lineage in the Tokyo group (log-rank test: *P* = 0.0003; GLMM: *z* = 2.105, *P* = 0.035; Fig. 5B). In other words, the high larval viability in the Tokyo group cannot be explained without introgression of introduced *B. japonicus japonicus*. Related to the above-mentioned demographic factors, the introduction of numerous nonnative toads and advancing habitat loss in urban Tokyo would have increased larval density and selection forces within the small ponds. As a result, a combination of these factors may have facilitated genotype displacement from native *B. japonicus formosus* to introduced *B. japonicus japonicus*.

In any case, we have to detect what led to the lower viability of *B. japonicus formosu*s in future studies. In mainland Japan, the cityscape is continuous right up to the mountain border and the problems of habitat loss and fragmentation are serious issues not only in Tokyo but also outside the Tokyo area (the Eastern group, particularly Pop. 5, has low larval viability [Table S1]). The similar high *F*_IS_ values for the Eastern and Tokyo populations (Table A2; 0.30 [Eastern] and 0.29 [Tokyo]) may indicate that a similar environment has increased the risk of inbreeding.

### Evolutionary study for urban ecology

The fitness of the Japanese common toad *B. japonicus* in urban Tokyo may be increasing with the assistance of nonnative individuals. At the same time, the toads have lost some of their nativeness, that is, some distinct local genetic components of the original populations. We would like to stress that although the high larval viability of *B. japonicus* in urban Tokyo may offer temporary remission, genetic disturbance and loss of originality are permanent consequences of their artificial introduction. The phenomena are ongoing in the urban toads, and more observational studies are required to determine the effects of these issues on an evolutionary time scale.

We anticipate that this study will draw the attention of evolutionary and conservation biologists to problems encompassing genetic disturbance and urbanization. It is desirable to take an approach that integrates urban ecology, conservation genetics, and evolutionary biology in the future to find solutions for problems associated with the cohabitation of humans and wild animals.

## Data Archiving Statement

Data for this study are available at Dryad - doi:10.5061/dryad.67r45

## Appendix

**Table A1:** Details of allele information and polymerase chain reaction (PCR) conditions for the microsatellite loci amplified in *Bufo japonicus*

Locus ID	Multiplex	Repeat unit	Annealing temperature (°C)	Extension temperature (°C)	Dye label	Allele size range (bp)/(in *B. bufo*)	No. of alleles /(in *B. bufo*)	*H*_O_/(in *B. bufo*)	*H*_E_/(in *B. bufo*)	GeneBank accession no.
*Bbufu*11	MP1	(CA)_19_	54_(3)_–52_(3)_–50_(3)_–48_(3)_–46_(23)_	72	D4	92–96	3	0.437	0.570	AY037809
						(90–122)	(10)	(0.333)	(0.785)	
*Bbufu*13	MP2	(CA)_14_	62_(3)_–60_(3)_–58_(19)_	72	D4	142–154	7	0.300	0.516	AY037810
						(115–145)	(7)	(0.731)	(0.630)	
*Bjap*14	MP2	(GT)_7_TTTGT	62_(3)_–60_(3)_–58_(19)_	72	D3	105–113	7	0.373	0.498	AB713497
						(172–182)	(2)	(0.375)	(0.468)	
*Bbufu*23	MP3	(AC)_19_	61_(3)_–59_(3)_–57_(3)_–55_(21)_	72	D4	112–122	8	0.529	0.715	AY037814
						(96–104)	(3)	(0.370)	(0.611)	
*Bbufu*39	MP1	(GT)_3_CTT(GT)_14_	54_(3)_–52_(3)_–50_(3)_–48_(3)_–46_(23)_	72	D2	182–196	8	0.304	0.478	AY037816
						(162–183)	(4)	(0.575)	(0.509)	
*Bbufu*49	MP3	(GT)_29_	61_(3)_–59_(3)_–57_(3)_–55_(21)_	72	D2	134–150	9	0.273	0.384	AY037819
						(144–178)	(17)	(0.513)	(0.916)	
*Bbufu*62	MP3	(GT)_18_	54_(3)_–52_(3)_–50_(3)_–48_(3)_–46_(23)_	72	D3	150–168	9	0.502	0.775	AY037821
						(195–227)	(7)	(0.625)	(0.703)	

Loci were amplified in two or three multiplex reactions (MP1, MP2, and MP3) using the Multiplex PCR Kit (Qiagen) and PCR conditions recommended by the manufacturer. One primer was labeled with fluorochrome for each locus (D4, D3, or D2 Beckman dyes). PCR was conducted in a GeneAmp PCR System 2700 (Applied Biosystems) thermocycler with a total reaction volume of 5 or 10 μL and was initiated with a 15 min denaturation period (95°C). Thermocycling included touchdown steps consisting of a 30 sec 94°C denaturation, a 90 sec primer-specific annealing with the temperature reduced in 2°C increments at each step (numbers in parentheses in the Annealing temperature column indicate the number of successive cycles at the specified temperature) and an extension step at 72°C for 1 min. All reactions culminated with a final extension at 60°C for 30 min.

**Table A2:** The population size (*N*), number of alleles, allelic richness (A), expected heterozygosity (H_E_), and the inbreeding coefficient within populations (F_IS_) for each local population across the 7 loci

Group	Pop. ID	*N*	No. alleles	A	H_E_	F_IS_
Eastern	1	31	5	1.50	0.57	**0.268**
	2	2	1.86	1.52	0.57	0.625
	3	15	3.29	1.66	0.48	**0.386**
	4	8	2.57	1.75	0.43	0.05
	5	14	3	1.85	0.40	−0.04
	12	1	–	–	–	–
	13	12	4.00	2.98	0.56	**0.364**
	14	10	2.43	2.99	0.41	−0.278
	15	2	2.00	3.21	0.57	–
	Total	95	6.57	6.17	0.57	**0.30**
Tokyo	6	25	3.29	2.04	0.47	**0.275**
	7	10	3.29	2.20	0.53	**0.402**
	8	15	2.43	2.23	0.41	**0.332**
	9	32	3.14	2.37	0.42	−0.015
	10	27	3.43	2.58	0.52	**0.26**
	11	24	3.43	2.73	0.55	**0.326**
	Total	133	5.0	4.65	0.512	**0.29**
Western	16	5	2.71	3.36	0.55	−0.046
	17	20	3.14	3.43	0.49	0.112
	18	32	3.71	3.53	0.46	**0.145**
	19	8	3.00	3.71	0.54	**0.218**
	Total	65	4.14	4.14	0.50	**0.163**

Values significantly different from zero are indicated in bold.

**Table A3:** AMOVA of 19 sites *Bufo japonicus* in three groups, Tokyo, Eastern, and Western

Source of variation	Degree of freedom	Sum of squares	% of variation
Among groups	2	73.86	**7.01**
Among populations within groups	16	117.71	**9.18**
Among individuals within populations	274	562.27	**17.05**
Total	292	753.84	

Significant values are in bold.

**Table A4:** Pairwise *F*_ST_ between populations

Pop ID	1	3	4	5	6	7	8	9	10	11	13	14	16	17	18
3	**0.11**														
4	**0.16**	**0.21**													
5	**0.14**	**0.21**	**0.25**												
6	**0.15**	**0.19**	**0.24**	**0.16**											
7	**0.08**	**0.16**	**0.19**	**0.17**	0.03				**Tokyo**						
8	**0.18**	**0.23**	**0.24**	**0.21**	**0.08**	0.06									
9	**0.18**	**0.25**	**0.27**	**0.22**	**0.07**	**0.09**	**0.08**								
10	**0.17**	**0.19**	**0.22**	**0.14**	**0.07**	**0.11**	**0.11**	**0.11**							
11	**0.15**	**0.20**	**0.22**	**0.14**	**0.04**	**0.05**	**0.11**	**0.09**	0.03						
13	0.06	**0.15**	**0.22**	**0.19**	**0.21**	**0.12**	**0.24**	**0.24**	**0.20**	**0.17**					
14	**0.08**	**0.12**	**0.26**	**0.12**	**0.12**	**0.11**	**0.26**	**0.23**	**0.16**	**0.15**	0.13				
16	**0.18**	**0.25**	0.32	**0.24**	**0.10**	0.05	**0.13**	**0.20**	**0.13**	**0.08**	**0.21**	0.24			
17	**0.20**	**0.21**	**0.23**	**0.21**	**0.11**	**0.14**	**0.12**	**0.07**	**0.11**	**0.11**	**0.23**	**0.23**	**0.16**		
18	**0.19**	**0.18**	**0.21**	**0.15**	**0.06**	**0.10**	**0.08**	**0.08**	**0.09**	**0.09**	**0.23**	**0.17**	**0.15**	0.02	
19	**0.16**	**0.23**	**0.28**	**0.22**	**0.08**	0.08	**0.10**	**0.06**	**0.08**	**0.06**	**0.20**	**0.22**	0.08	0.02	0.07

Significant values are in bold.

## Supporting Information

**Figure S1.** Additional data for the Bayesian clustering method implemented in STRUCTURE (Pritchard et al. 2000): (A) the relationship between ΔK (likelihoods) and *K* (= the number of genetic clusters). (B) Graphical output from STRUCTURE for *K* = 3 based on the seven microsatellite markers for *Bufo japonicus*. Population ID is corresponding to the number of Figure 3.

**Table S1.** Information on monitoring of larval survivorship.
